# Diagnostic, Prognostic, and Predictive Tissue Biomarkers in Urothelial Carcinoma In Situ: A Narrative Review

**DOI:** 10.3390/diagnostics15172163

**Published:** 2025-08-26

**Authors:** Francesca Sanguedolce, Angelo Cormio, Magda Zanelli, Maurizio Zizzo, Andrea Palicelli, Ugo Giovanni Falagario, Giulio Milanese, Andrea Benedetto Galosi, Roberta Mazzucchelli, Luigi Cormio, Giuseppe Carrieri

**Affiliations:** 1 Pathology Unit, Policlinico Foggia, University of Foggia, 71122 Foggia, Italy; 2 Department of Urology, Azienda Ospedaliero-Universitaria Ospedali Riuniti Di Ancona, Università Politecnica Delle Marche, Via Conca 71, 60126 Ancona, Italy; g.milanese@univpm.it (G.M.); a.b.galosi@univpm.it (A.B.G.); 3 Department of Urology and Renal Transplantation, Policlinico Foggia, University of Foggia, 71122 Foggia, Italy; ugo.falagario@unifg.it (U.G.F.); luigi.cormio@unifg.it (L.C.); giuseppe.carrieri@unifg.it (G.C.); 4 Pathology Unit, Azienda USL-IRCCS di Reggio Emilia, 42123 Reggio Emilia, Italy; magda.zanelli@ausl.re.it (M.Z.); andrea.palicelli@ausl.re.it (A.P.); 5 Surgical Oncology Unit, Azienda USL-IRCCS di Reggio Emilia, 42123 Reggio Emilia, Italy; maurizio.zizzo@ausl.re.it; 6 Clinical and Experimental Medicine PhD Program, University of Modena and Reggio Emilia, 41121 Modena, Italy; 7 Urology Unit, AST 5, Via degli Iris 1, 63100 Ascoli Piceno, Italy; 8 Section of Pathological Anatomy, Department of Biomedical Sciences and Public Health, United Hospitals, Università Politecnica delle Marche, 60126 Ancona, Italy; r.mazzucchelli@univpm.it; 9 Department of Urology, Bonomo Teaching Hospital, 76123 Andria, Italy

**Keywords:** urothelial carcinoma in situ, bladder cancer, bacillus Calmette-Guérin, biomarkers, immunohistochemistry, CK20, CD44, p53, Ki-67, PD-L1, nectin-4

## Abstract

Urothelial carcinoma in situ (UCIS) is a high-grade non-muscle-invasive neoplasm with significant clinical implications due to its potential for progression to muscle-invasive disease. Accurate diagnosis and risk stratification are crucial for appropriate management, particularly given the variability in response to intravesical Bacillus Calmette-Guérin (BCG) therapy. While the diagnosis of UCIS primarily relies on morphological criteria, immunohistochemical (IHC) markers serve as valuable ancillary tools, particularly in challenging cases. Markers such as CK20, CD44, p53, and Ki-67 have been extensively studied, though none demonstrate complete sensitivity or specificity. Additionally, molecular classification has identified luminal and basal subtypes, with potential prognostic and therapeutic implications. Recent studies have also explored predictive biomarkers for BCG response, including PD-L1, whose expression correlates with recurrence and potential responsiveness to immune checkpoint inhibitors. Emerging targeted therapies, such as enfortumab vedotin, have shown promise, with nectin-4 overexpression observed in most UCIS cases. Despite these advancements, challenges remain, including interobserver variability in morphological assessment, heterogeneous IHC methodologies, and the need for standardized molecular testing. This review highlights the current understanding of diagnostic, prognostic, and predictive tissue biomarkers in UCIS, underscoring the potential role of molecular profiling in guiding personalized treatment strategies. Future research should focus on refining biomarker-driven classification systems to improve risk stratification and therapeutic decision-making in UCIS patients.

## 1. Introduction

Urothelial carcinoma in situ (UCIS) is the only flat neoplastic lesion formally recognized as a distinct pathological entity in the fifth edition of the World Health Organization (WHO) classification of genitourinary tumors. It is characterized by marked cytological atypia, comparable in severity to that typically seen in high-grade papillary urothelial carcinoma (UC) [1]. UCIS is defined as a malignant, non-invasive flat lesion that is confined to the urothelium and does not penetrate the underlying lamina propria. From a clinical standpoint, UCIS falls under the category of non-muscle invasive urothelial carcinomas and, as such, is amenable to conservative management strategies, most notably intravesical therapy with bacillus Calmette–Guérin (BCG) instillations. In particular, UCIS is considered a high-risk lesion for progression, as more than 50% of patients who do not receive appropriate treatment go on to develop muscle-invasive UC, which is associated with a significantly worse prognosis [2].

Moreover, under the current European Association of Urology (EAU) risk stratification model, all patients diagnosed with UCIS are classified as either high risk or very high risk, underscoring the aggressive biological potential of this entity and the importance of timely therapeutic intervention [3]. For all these reasons, accurate and early histopathological diagnosis, along with appropriate treatment selection, is crucial for optimal patient outcomes [4].

**Figure 1:**
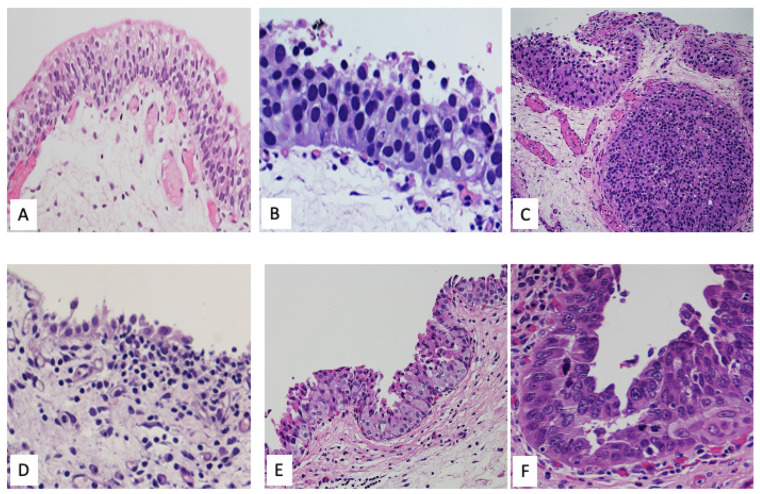
(**A**): Normal urothelium, (**B**): UCIS: mitotic figures throughout the entire thickness of the urothelium, (**C**): UCIS involving von Brunn nest, (**D**): UCIS denuding variant, (**E**): UCIS with pagetoid pread, (**F**): UCIS large cell variant.

The essential histological criteria for diagnosing UCIS on transurethral resection or biopsy specimens include the identification of urothelial cells exhibiting unequivocal high-grade malignant features. These include pronounced nuclear abnormalities, such as increased nuclear-to-cytoplasmic ratio, nuclear enlargement, hyperchromasia, membrane irregularities, and nuclear pleomorphism. Additionally, architectural disorganization of the urothelium is typically present, with features such as loss of cellular polarity, crowding, and disordered maturation. These morphological alterations are often appreciable at low to medium magnification and are seen in the absence of papillary structures or stromal invasion, which is critical to distinguish UCIS from other flat or exophytic lesions [5,6] (Figure 1 and Figure S1).

UCIS can show various cytomorphological patterns, including large cell, small cell, plasmacytoid, pagetoid, and denuding cystitis/clinging patterns. These patterns do not have significant clinical implications [5,7], but may pose diagnostic challenges, and more than one pattern can be present simultaneously in the same case [8]. Like their invasive counterpart, rare cases of CIS may exhibit squamous and glandular differentiation, the latter showing CDX2 expression accordingly [8,9,10].

This article was conceived as a narrative review, which, by definition, does not follow a formal systematic search strategy or predefined inclusion/exclusion criteria. In narrative reviews, literature selection is typically guided by the authors’ expertise, knowledge of the field, and the intended scope of the manuscript, rather than by a structured, reproducible search process [11,12]. Accordingly, we integrated key and recent publications deemed most relevant to the topic.

## 2. Diagnostic Markers

**Figure 2:**
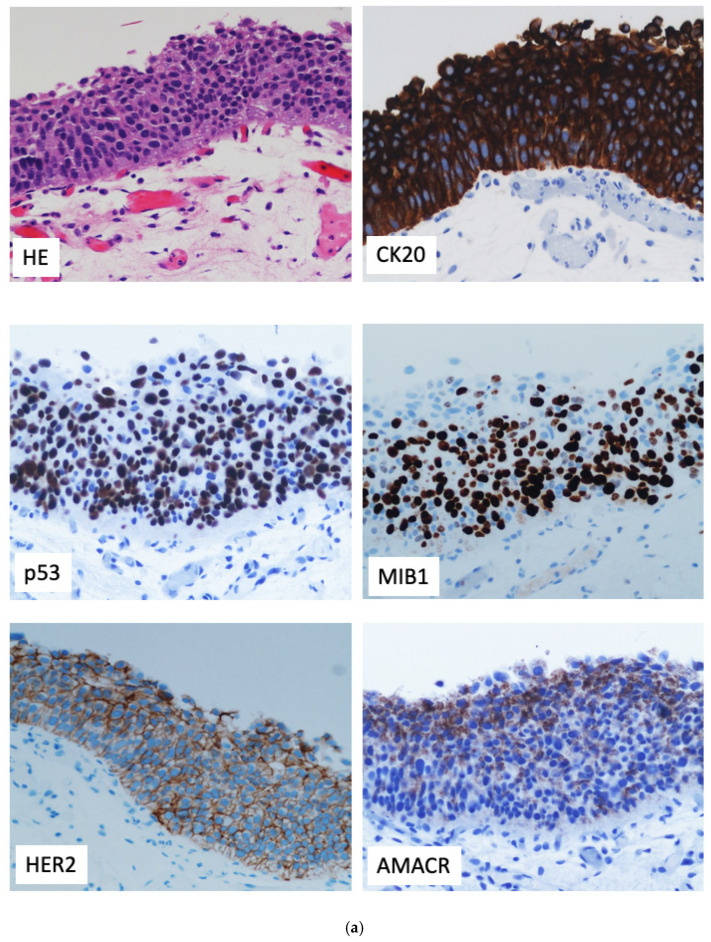

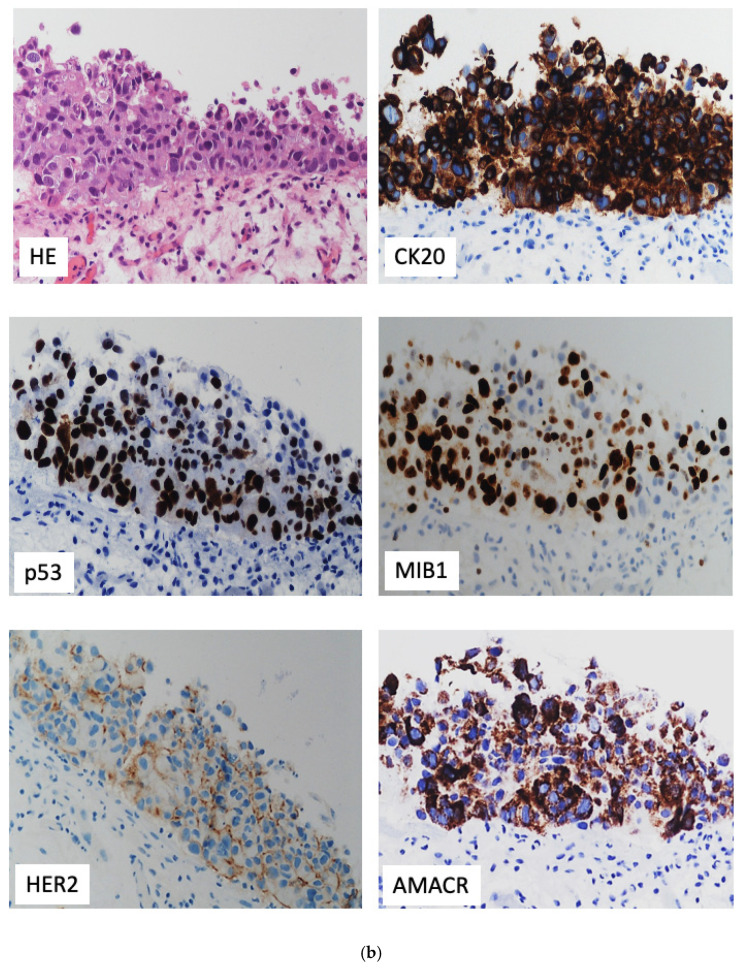
(**a**,**b**). Representative images of 2 cases of UCIS: haematoxylin-eosin and selected immunohistochemical markers (see text). Note variable staining of AMACR and HER2.

The diagnosis of flat urothelial lesions with atypia—including reactive atypia, urothelial dysplasia, and carcinoma in situ of the urothelium (UCIS)—is primarily and fundamentally based on the morphological features observed in hematoxylin-eosin-stained histological slides, hence, the evaluation relies on architectural patterns and cytologic abnormalities identified under light microscopy. However, in diagnostically challenging cases or in case of ambiguous morphological features, immunohistochemistry (IHC) may serve as a useful ancillary tool to provide additional support to the interpretation. Despite this potential aid, the routine use of IHC is not generally recommended, as no individual immunohistochemical marker currently available has demonstrated both complete sensitivity and absolute specificity in distinguishing among these lesions [13,14]. For this reason, the role of IHC should be considered complementary—an adjunct to, rather than a substitute for, morphological assessment that is strongly suggestive of UCIS. When applied in isolation or in cases without clear morphological context, IHC results can be misleading, as considerable immunophenotypic overlap may occur among reactive, dysplastic, and neoplastic conditions [6,8,15] (Figure 2a,b).

The availability of limited tissue in bladder biopsies makes the application of molecular techniques more challenging than IHC in this setting. However, in a recent study on 13 cases of small/flat atypical urothelial lesions, the authors achieved optimal results using next generation sequencing (NGS) testing with appropriate methodology. Nevertheless, the limited availability of molecular techniques in routine diagnostics and their higher costs mean that they can only be considered in selected cases [16]. The use of certain markers, alone or in combination, can be helpful in the differential diagnosis of atypical flat urothelial lesions and in the diagnosis of UCIS in specific cases, such as in the post-treatment setting and in less common morphological patterns [5,9,13,17,18,19]. As reported in a recent study, the markers currently used in the differential diagnosis between benign and malignant flat urothelial lesions in the bladder may not be reliable in samples from the upper urinary tract, possibly due to intrinsic biological and anatomical characteristics [20].

### 2.1. CK20

CK20, an epithelial intermediate filament, is usually present in a subset of normal tissues, including gastrointestinal epithelium and Merkel cells [21], is frequently used in the evaluation of flat bladder lesions. In normal and benign reactive urothelium, CK20 expression is restricted to the superficial umbrella cells, where it plays a crucial role in maintaining urothelial lining integrity and providing the elasticity needed to accommodate daily fluctuations in urine volume [22]. Conversely, in dysplasia and UCIS, CK20 is expressed in the intermediate and basal cell layers and/or throughout the entire urothelial thickness in up to 90% of cases [23], often with staining intensity increasing in parallel with the severity of the lesion (from dysplasia to CIS). According to a recent meta-analysis and diagnostic test accuracy review [24], CK20 and CD44 showed higher sensitivity and specificity compared to p53 and AMACR, although this does not preclude the latter’s potential diagnostic role in specific contexts.

### 2.2. P53

P53 is frequently used in this setting [24,25,26]. The p53 protein is encoded by a tumor suppressor gene located on chromosome 17, involved in transcriptional regulation, and is frequently mutated or otherwise altered in UCIS [10]. In normal and reactive urothelium, p53 typically exhibits a wild-type pattern characterized by nuclear expression of heterogeneous extent and intensity, predominantly in the basal/parabasal layers. In dysplasia and UCIS, p53 is either strongly and diffusely expressed or completely absent (null phenotype), as a consequence of gene mutations [13]. On the other hand, only up to 60% of UCIS cases harbor TP53 mutations and exhibit abnormal p53 expression by IHC [13,15,27], although the specificity of this marker can be up to 100% [15]. As a standalone marker, p53 can be misleading, particularly in the differential diagnosis between UCIS and radiation-induced atypia, since in the latter setting p53 expression appears increased compared to other forms of reactive atypia [21]. A particularly challenging differential diagnosis is posed by viral infections, due to the presence of significant cytologic atypia. These cells, whose nuclear details are often better appreciated in cytologic preparations than in histologic sections, may show strong p53 positivity and a high Ki-67 proliferative index. In cases where the clinical context supports a diagnosis of BK virus infection, the use of a specific marker—namely, an antibody against SV40 (simian virus 40 polyomavirus)—can reveal the presence of infected cells. However, this does not completely rule out the diagnosis of carcinoma, as an association between BK virus infection and an increased risk of urothelial carcinoma has been documented [28]. The conflicting results reported in the literature may be related to differences in interpretative criteria. While many studies have dichotomized cases into aberrant expression (diffuse, strong, or null) versus normal/wild-type [15] other authors have opted to calculate the percentage of positive cells [29], or used semiquantitative scoring systems [30]. Aberrant p53 expression (particularly strong and diffuse nuclear staining), combined with a high Ki-67 proliferative index, has been reported as a key factor in the differential diagnosis between the ‘overriding’ form of UCIS and normal umbrella cells [31]. Likewise, immunocytochemical staining for p53 and Ki-67 is useful for improving diagnostic accuracy in the detection of UC cells in urine cytology [32]. Interestingly, a study tested a cohort of flat urothelial lesions using dual immunohistochemical staining for p53 and CK20 to identify cells co-expressing both markers. The percentage of these double-positive cells proved to be a statistically significant parameter in the differential diagnosis between UCIS and other lesions (reactive urothelial atypia, atypia of unknown significance, and urothelial dysplasia) [29]. Accordingly, the CK20-negative cases identified in the cohort by Lombardo et al. did not exhibit abnormal p53 expression or a high Ki-67 proliferative index [33].

### 2.3. CD44

CD44, a transmembrane adhesion molecule that interacts with extracellular hyaluronan and links to intracellular cytoskeletal structures, is considered a useful marker in the differential diagnosis of UCIS versus other flat urothelial lesions. It plays various physiological roles in the urothelium, including cell adhesion, lymphocyte homing/activation, and cell motility [34]. Its expression pattern is inverse to that of CK20, as in benign urothelium, CD44 shows cytoplasmic staining restricted to the basal/parabasal layer [24], whereas in UCIS, particularly the pagetoid type, and in reactive atypia, CD44 shows complete absence or significant reduction (such as, patchy staining in basal cells), and full-thickness staining with membrane accentuation, respectively [13]. The fact that high CD44 expression has been reported in normal/reactive urothelium in a recent meta-analysis makes it a useful negative marker in the differential diagnosis of UCIS, with a sensitivity and specificity of 0.865 and 0.767, respectively, according to the diagnostic test accuracy analysis [35]. Aberrant phenotypes have been reported in selected cases. For example, strong CD44 expression is more commonly observed in plasmacytoid UCIS (approximately 30% of cases) and in a subset of UCIS cases presumed to be of the ‘basal’ type (see below) [36,37]. However, the results obtained in previous studies are significantly affected by the technical difficulties historically reported in achieving reliable staining, which are now generally overcome through methodological standardization.

### 2.4. Ki-67

Ki-67, a marker of actively proliferating cells, is typically high with full-thickness distribution in urothelial dysplasia/UCIS, whereas in normal urothelium, it is low and restricted to the basal and suprabasal layers (up to 10% of basal cells) [13,25,26]. In routine practice, Ki-67 immunoreactivity is most commonly assessed using the MIB-1 monoclonal antibody, which targets the Ki-67 nuclear antigen.

Although it has been reported that reactive atypia is not associated with a consistent increase in proliferative index, but rather with a suprabasal localization of nuclear staining [13], a significant overlap in Ki-67 staining has been reported between UCIS and reactive urothelial atypia [15]. This finding, along with the potential for false positives (such as inflammatory cells migrating into the urothelium from the lamina propria), makes Ki-67 less useful in this context [13], and its routine use is generally not recommended [5].

### 2.5. AMACR

Alpha-methylacyl-CoA racemase (AMACR) is another useful marker. It is typically positive in urothelial dysplasia/UCIS but negative in reactive/benign urothelium, although the absence of staining does not completely rule out UCIS [38]. On the other hand, AMACR staining is not affected by potential false positives that may be observed with CK20 in normal urothelium. This issue arises when a tangential section of a transurethral biopsy or resection specimen includes only umbrella cells and the immediately underlying ones, which may exhibit full-thickness CK20 expression, potentially leading to a misdiagnosis of UCIS [27,39]. However, some authors have reported that even weak AMACR expression in the superficial cells of normal urothelium can be observed [38,40,41]. As compared to CK20, AMACR has been found to be less sensitive but more specific, with the same issue of weaker staining intensity [27,40]. Interestingly, some studies have reported differences in sensitivity depending on the presence or absence of prior treatment in the examined cases [38]. In a recent meta-analysis, Yoo et al. analyzed the expression rates of CK20, CD44, AMACR, and p53 in UCIS and their respective diagnostic accuracy. In the same meta-analysis [35], AMACR was the only marker showing a statistically significant odds ratio for differentiating UCIS from normal/reactive urothelium, despite CK20 and CD44 achieving higher pooled sensitivity and specificity values.

### 2.6. Other Markers and Panels

Several studies have described strong, full-thickness HER2 expression in UCIS, with some series reporting this pattern in the majority of cases [42,43], while others found overexpression in approximately one-third [44]. This variability likely reflects differences in sample size, antibody clones, and scoring criteria across studies. Interestingly, across multiple studies, HER2 demonstrated greater specificity for CIS than other commonly used markers, such as CK20, CK5/6, P53, and CD138 [42,45].

PRAME (Preferentially Expressed Antigen in Melanoma) is a cancer/testis antigen that has proven useful in recent years, particularly in the diagnostic assessment of melanocytic lesions. The limited data available in the literature on PRAME expression in UCIS indicate low expression levels, ranging from 7% to 20% [46,47,48]. Fujii et al. reported PRAME expression in 43.4% of their cohort of 53 UCIS cases, demonstrating its limited effectiveness as a diagnostic marker [4]. However, as a potential target for immunotherapeutic drugs, PRAME could be useful in identifying UCIS patients who may benefit from such treatment.

The expression of claspin, a nuclear protein associated with DNA replication and damage response, has been studied in a cohort of UCIS cases. In this study, claspin staining was weak or absent in most samples of benign urothelium but strong in UCIS samples, with a specificity as high as 96% [49].

In recent years, some studies have applied antibody cocktails containing two or three markers to UCIS cases, achieving different detection colors and complementary expression patterns [34,38]. Arias-Stella et al. tested CK20 and p53 in a cohort of 69 cases with equivocal urothelial atypia, reporting conflicting results, with a predominant proportion of discordant (26%) and indeterminate (45%) staining patterns. As a result, clinical data proved to be prognostically superior to the immunophenotype [50]. In this setting, Aron et al. reported high sensitivity—superior to that of individual markers—of the malignant IUN-3 cocktail immunoreactivity pattern (CK20 and/or p53 full-thickness expression with negative CD44) in distinguishing UCIS from reactive urothelial atypia, both in pretherapy and posttherapy settings [38]. The same panel, applied to a series of 127 bladder biopsies diagnosed as reactive atypia, atypia of uncertain significance, low-grade dysplasia, or UCIS by general pathologists, was shown to enhance diagnostic accuracy, particularly in distinguishing atypia of uncertain significance and low-grade dysplasia from UCIS. Meanwhile, it was able to confirm UCIS cases diagnosed based on morphological criteria alone, in the post-treatment setting as well [51]. A few years ago, our group proposed a diagnostic algorithm to improve the classification of flat urothelial lesions, whose histological and immunophenotypical overlap can complicate diagnostic interpretation. The algorithm was designed to integrate morphological assessment with immunohistochemical (IHC) profiling, aiming to improve diagnostic accuracy in differentiating reactive atypia, atypia of unknown significance (AUS), dysplasia, and UCIS [18]. We reviewed and summarized the WHO 2016 [18] classification updates and evaluated a panel of IHC markers—including CK20, p53, CD44, Ki-67, and p16—based on their expression profiles across the spectrum of flat urothelial lesions. The diagnostic strategy begins with morphological evaluation and selectively incorporates IHC when the histologic and clinical context is ambiguous. Our findings supported the use of a triplet panel (CK20, p53, CD44), with possible adjuncts, to enhance reproducibility and interobserver agreement.

### 2.7. UTUC-Specific Considerations

The immunohistochemical thresholds and patterns described for UCIS of the bladder may not be directly applicable to upper tract urothelial carcinoma (UTUC). Differences in urothelial thickness, exposure to urine, and background inflammation can influence staining intensity and distribution. A recent study [20] reported that CK20, CD44, and p53 patterns used for bladder lesions showed lower diagnostic reliability in UTUC, suggesting that interpretation should be made with caution and in the appropriate morphological context.

## 3. Molecular Classification

Similarly to muscle-invasive UC, within the molecular classification of UCS, lesions classified as UCIS can exhibit luminal or basal expression patterns [44,46]. Primary (de novo) UCIS tends to cluster within so-called ‘infiltrated luminal expression patterns’ [52], whereas cases of muscle-invasive UC with concomitant UCIS (secondary UCIS) may fall into the ‘basal-squamous’ (basal-like) classification, as defined in the work of Robertson et al. [53]. Using a representative immunohistochemical panel, a large cohort study (*n* = 156) demonstrated that approximately 85% of UCIS cases express luminal markers (CK20, GATA3, and ER-β), one-third overexpress HER2, and only a few cases exhibit basal marker expression (CK5/CK6) [44]. The predominance of the luminal phenotype (CK20+/GATA3+/CK5/6−/CK14−) in UCIS cases, particularly in solitary lesions, has been confirmed in subsequent studies [54]. These studies have also highlighted the importance of interlesional and intralesional heterogeneity in the evaluation of immunohistochemical surrogates for molecular classification [55,56]. Currently, the prognostic impact of using these markers to stratify patients with UCIS has not been unequivocally proved [54]. It has been suggested that ‘basal/squamous’ immunohistochemical markers may effectively support the identification of the minor subset of UCIS with aberrant overexpression of CK5, CK14, and CD44 [37,42,57,58,59]. Interestingly, a phenotypic study on paired UCIS and invasive UC from the same biopsy demonstrated a significant loss of luminal marker expression during progression, while an increase in basal marker expression in the invasive compartment suggests that luminal-type UCIS undergoes a class switch to the basal type during progression [44]. Understanding these phenotypic differences in relation to molecular classification is crucial not only from a prognostic perspective but also as a key to interpreting cases where the immunohistochemical staining of a single marker does not align with morphology.

## 4. Prognostic/Predictive Markers

The lack of responsiveness to BCG therapy is common and represents a crucial parameter in deciding whether to treat UCIS with radical surgery [60]. Approximately 10–20% of patients who respond and 66% of those who do not respond progress to muscle-invasive disease [61,62,63]. Moreover, 15–25% of patients undergoing cystectomy for UCIS present with microinvasive disease. The prognosis of UCIS varies based on multiple factors, mostly being clinical parameters such as disease extent (focal, multifocal, or extensive), involvement of the prostatic urethra, and response to therapy [64]. Therefore, biomarkers predicting BCG responsiveness are of particular interest. Programmed death-ligand 1 (PD-L1) expression has been preferentially identified in patients with UCIS who experience recurrence after BCG in several studies [33,65,66]. However, PD-L1 is not currently a validated predictive biomarker for BCG response in clinical practice. Combined intravesical BCG and PD-(L)1 blockade remains exploratory and is being investigated in early-phase clinical trials [67]. Some studies have reported a prognostic association between p53 overexpression and disease progression [68,69], though the prognostic role of p53 may be supported by more complex mechanisms. Shariat and colleagues [70] reported a frequent association between p53 and p21 positivity in a cohort of CIS cases, the latter being is a cyclin-dependent kinase inhibitor involved in cell cycle regulation; however, only p21 expression was associated with risk of progression to invasive carcinoma. More recently, other authors have suggested that p53 may play a role in the pathogenesis of UCIS, stratifying cases into basal and luminal subgroups that could have prognostic relevance [71]. Nevertheless, previous studies have failed to demonstrate a significant association between p53 expression and treatment response in CIS patients [71,72].

As mentioned above, the data supporting the prognostic role of molecular classification in non muscle invasive UC, and particularly in UCIS, are conflicting. However, it is noteworthy that Lombardo et al. reported that the absence of CK20 expression in UCIS (i.e., putative ‘basal-like’ lesions) is associated with a significantly higher rate of progression and similar recurrence rates after BCG therapy as compared to CK20-positive cases [33]. New targeted therapies have been emerging in uro-oncology in recent years. Nectin-4 is an immunoglobulin-like adhesion molecule and the target of the antibody-drug conjugate (ADC) enfortumab vedotin (EV). Preliminary evidence suggests high Nectin-4 expression in UCIS [73], but data are limited to small case series. Moreover, clinical applicability in CIS is yet to be established, considering intravesical delivery challenges and the potential toxicity profile of systemic ADC therapy.

## 5. Conclusions

Despite several intrinsic issues in this context (including the subjectivity of morphological assessment in the differential diagnosis between UCIS and other atypical flat urothelial lesions, heterogeneity in immunohistochemical methodologies and markers used across studies, with varying cut-offs and interpretative criteria, as well as relatively small cohort sizes in most studies), immunohistochemical markers remain the most commonly used ancillary tool to support morphology in the identification of UCIS in challenging cases. In this setting, our advice, based on literature findings, is to use panels of antibodies, and evidence-based algorithms which can assist pathologists in making more standardized and clinically relevant diagnoses in routine bladder biopsy specimens. Moreover, as UCIS is a potentially aggressive entity, immunohistochemical markers represent a promising perspective in the prognostic stratification and therapeutic management of these patients.

## Supplementary Materials

The following supporting information can be downloaded at: https://www.mdpi.com/article/10.3390/diagnostics15172163/s1, Figure S1: HE-stained histological section and diagram.

## Abbreviations

The following abbreviations are used in this manuscript:

UCIS	Urothelial carcinoma in situ
BCG	Bacillus Calmette-Guérin
IHC	immunohistochemical
NGS	next generation sequencing
AMACR	Alpha-methylacyl-CoA racemase
PRAME	Preferentially Expressed Antigen in Melanoma
EV	enfortumab vedotin
ADC	antibody-drug conjugate

## References

[1] Riddle N., Parkash V., Guo C.C., Shen S.S., Perincheri S., Ramirez A.S., Auerbach A., Belchis D., Humphrey P.A. (2024). Recent Advances in Genitourinary Tumors: Updates From the 5th Edition of the World Health Organization Blue Book Series. Arch. Pathol. Lab. Med..

[2] Lamm D.L. (1992). Carcinoma in situ. Urol. Clin. N. Am..

[3] European Association of Urology (EAU) (2024). EAU Guidelines. Proceedings of the EAU Annual Congress Paris 2024 Ed..

[4] Fujii S., Ishida M., Komura K., Nishimura K., Tsujino T., Saito T., Taniguchi Y., Murakawa T., Azuma H., Hirose Y. (2023). Expression of Preferentially Expressed Antigen in Melanoma, a Cancer/Testis Antigen, in Carcinoma In Situ of the Urinary Tract. Diagnostics.

[5] McKenney J.K. (2021). Urothelial carcinoma in situ: Diagnostic update. Pathology.

[6] Netto G.J.T.T., Compérat E.M., Williamson S.R., Board WCoTE (2022). Urothelial carcinoma in situ. Urinary and Male Genital Tumours.

[7] Compérat E., Jacquet S.F., Varinot J., Conort P., Roupret M., Chartier-Kastler E., Bitker M.O., Witjes J.A., Cussenot O. (2013). Different subtypes of carcinoma in situ of the bladder do not have a different prognosis. Virchows Arch..

[8] Compérat E., Kläger J., Oszwald A., Shariat S., Wasinger G. (2024). How to distinguish between reactive and neoplastic flat urothelial lesions. Diagn. Histopathol..

[9] Chan T.Y., Epstein J.I. (2001). In situ adenocarcinoma of the bladder. Am. J. Surg. Pathol..

[10] Wang G., Zhou H., Guo C.C., Ro J.Y. (2021). Flat Urothelial Lesions. Urinary Bladder Pathology.

[11] Ferrari R. (2015). Writing narrative style literature reviews. Med. Writ..

[12] Grant M.J., Booth A. (2009). A typology of reviews: An analysis of 14 review types and associated methodologies. Health Inf. Libr. J..

[13] Li J., Wilkerson M.L., Deng F.M., Liu H. (2024). The Application and Pitfalls of Immunohistochemical Markers in Challenging Diagnosis of Genitourinary Pathology. Arch. Pathol. Lab. Med..

[14] McIntire P., Khan R., Kilic I., Wojcik E.M., Pambuccian S.E., Barkan G.A. (2019). Immunohistochemistry in the workup of bladder biopsies: Frequency, variation and utility of use at an academic center. Ann. Diagn. Pathol..

[15] Nguyen J.K., Przybycin C.G., McKenney J.K., Magi-Galluzzi C. (2020). Immunohistochemical staining patterns of Ki-67 and p53 in florid reactive urothelial atypia and urothelial carcinoma in situ demonstrate significant overlap. Hum. Pathol..

[16] Pinard A., Chen C., Van Ziffle J., Simko J.P., Stohr B.A., Chan E. (2024). Next-generation sequencing has diagnostic utility in challenging small/flat urothelial lesions. Ann. Diagn. Pathol..

[17] Amin M.B., Trpkov K., Lopez-Beltran A., Grignon D. (2014). Best practices recommendations in the application of immunohistochemistry in the bladder lesions: Report from the International Society of Urologic Pathology consensus conference. Am. J. Surg. Pathol..

[18] Sanguedolce F., Brunelli M., D’Amuri A., Calò B., Mancini V., Carrieri G., Cormio L. (2018). Evolving concepts and use of immunohistochemical biomarkers in flat non-neoplastic urothelial lesions: WHO 2016 classification update with diagnostic algorithm. Biomarkers.

[19] Sangoi A.R., Chan E., Abdulfatah E., Stohr B.A., Nguyen J., Trpkov K., Siadat F., Hirsch M., Falzarano S., Udager A.M. (2022). p53 null phenotype is a “positive result” in urothelial carcinoma in situ. Mod. Pathol..

[20] Irwin T., Donlan A.W., Owens L., Alvarez R., Vakar-Lopez F., Tretiakova M. (2024). Enhancing upper tract urothelial carcinoma diagnosis: Utility of cytokeratin 17 and CK20/CD44/p53 immunohistochemical panel. Hum. Pathol..

[21] Oliva E., Pinheiro N.F., Heney N.M., Kaufman D.S., Shipley W.U., Gurski C., Spicer B., Paner G.P., Gown A.M., Amin M.B. (2013). Immunohistochemistry as an adjunct in the differential diagnosis of radiation-induced atypia versus urothelial carcinoma in situ of the bladder: A study of 45 cases. Hum. Pathol..

[22] Alonso A., Ikinger U., Kartenbeck J. (2009). Staining patterns of keratins in the human urinary tract. Histol. Histopathol..

[23] Sanguedolce F., Russo D., Calò B., Cindolo L., Carrieri G., Cormio L. (2019). Diagnostic and prognostic roles of CK20 in the pathology of urothelial lesions. A systematic review. Pathol. Res. Pract..

[24] McKenney J.K., Desai S., Cohen C., Amin M.B. (2001). Discriminatory immunohistochemical staining of urothelial carcinoma in situ and non-neoplastic urothelium: An analysis of cytokeratin 20, p53, and CD44 antigens. Am. J. Surg. Pathol..

[25] Mallofré C., Castillo M., Morente V., Solé M. (2003). Immunohistochemical expression of CK20, p53, and Ki-67 as objective markers of urothelial dysplasia. Mod. Pathol..

[26] Kunju L.P., Lee C.T., Montie J., Shah R.B. (2005). Utility of cytokeratin 20 and Ki-67 as markers of urothelial dysplasia. Pathol. Int..

[27] Neal D.J., Amin M.B., Smith S.C. (2020). CK20 versus AMACR and p53 immunostains in evaluation of Urothelial Carcinoma in Situ and Reactive Atypia. Diagn. Pathol..

[28] Ross J., Li G., Yang X.J. (2020). Application and Pitfalls of Immunohistochemistry in Diagnosis of Challenging Genitourinary Cases. Arch. Pathol. Lab. Med..

[29] Di Sciascio L., Ambrosi F., Franceschini T., Giunchi F., Franchini E., Massari F., Bianchi F.M., Colecchia M., Fiorentino M., Ricci C. (2022). Could double stain for p53/CK20 be a useful diagnostic tool for the appropriate classification of flat urothelial lesions?. Pathol. Res. Pract..

[30] Yildiz I.Z., Recavarren R., Armah H.B., Bastacky S., Dhir R., Parwani A.V. (2009). Utility of a dual immunostain cocktail comprising of p53 and CK20 to aid in the diagnosis of non-neoplastic and neoplastic bladder biopsies. Diagn. Pathol..

[31] Bahceci D., Nguyen J.K., Sangoi A.R., Stohr B.A., Chan E. (2023). Urothelial carcinoma in situ with “overriding” features can evade detection by mimicking umbrella cells. Hum. Pathol..

[32] Courtade-Saïdi M., Aziza J., d’Aure D., Bérard E., Evrard S., Basset C., Lacoste-Collin L. (2016). Immunocytochemical staining for p53 and Ki-67 helps to characterise urothelial cells in urine cytology. Cytopathology.

[33] Lombardo K.A., Murati Amador B., Parimi V., Hoffman-Censits J., Choi W., Hahn N.M., Kates M., Bivalacqua T.J., McConkey D., Hoque M.O. (2021). Urothelial Carcinoma In Situ of the Bladder: Correlation of CK20 Expression With Adaptive Immune Resistance, Response to BCG Therapy, and Clinical Outcome. Appl. Immunohistochem. Mol. Morphol..

[34] Arville B., O’Rourke E., Chung F., Amin M., Bose S. (2013). Evaluation of a triple combination of cytokeratin 20, p53 and CD44 for improving detection of urothelial carcinoma in urine cytology specimens. CytoJournal.

[35] Yoo D., Min K.W., Pyo J.S., Kim N.Y. (2023). Diagnostic Roles of Immunohistochemical Markers CK20, CD44, AMACR, and p53 in Urothelial Carcinoma In Situ. Medicina.

[36] Sangoi A.R., Falzarano S.M., Nicolas M., McKenney J.K. (2019). Carcinoma In Situ With Plasmacytoid Features: A Clinicopathologic Study of 23 Cases. Am. J. Surg. Pathol..

[37] Mai K.T., Busca A., Belanger E.C. (2017). Flat Intraurothelial Neoplasia Exhibiting Diffuse Immunoreactivity for CD44 and Cytokeratin 5 (Urothelial Stem Cell/Basal Cell Markers): A Variant of Intraurothelial Neoplasia Commonly Associated With Muscle-invasive Urothelial Carcinoma. Appl. Immunohistochem. Mol. Morphol..

[38] Aron M., Luthringer D.J., McKenney J.K., Hansel D.E., Westfall D.E., Parakh R., Mohanty S.K., Balzer B., Amin M.B. (2013). Utility of a triple antibody cocktail intraurothelial neoplasm-3 (IUN-3-CK20/CD44s/p53) and α-methylacyl-CoA racemase (AMACR) in the distinction of urothelial carcinoma in situ (CIS) and reactive urothelial atypia. Am. J. Surg. Pathol..

[39] Akgul M., MacLennan G.T., Cheng L. (2020). The applicability and utility of immunohistochemical biomarkers in bladder pathology. Hum. Pathol..

[40] Alston E.L.J., Zynger D.L. (2019). Does the addition of AMACR to CK20 help to diagnose challenging cases of urothelial carcinoma in situ?. Diagn. Pathol..

[41] Fellegara G., Gabba S., Dorji T., De Luca G., Colecchia M. (2014). Observations on Aron et al’s “Utility of a triple antibody cocktail intraurothelial neoplasm-3 (IUN-3 CK20/CD44s/p53) and α-methylacyl-CoA racemase (AMACR) in the distinction of urothelial carcinoma in situ (CIS) and reactive urothelial atypia. Am. J. Surg. Pathol..

[42] Jung S., Wu C., Eslami Z., Tanguay S., Aprikian A., Kassouf W., Brimo F. (2014). The role of immunohistochemistry in the diagnosis of flat urothelial lesions: A study using CK20, CK5/6, P53, Cd138, and Her2/Neu. Ann. Diagn. Pathol..

[43] Sanguedolce F., Zanelli M., Palicelli A., Bisagni A., Zizzo M., Ascani S., Pedicillo M.C., Cormio A., Falagario U.G., Carrieri G. (2023). HER2 Expression in Bladder Cancer: A Focused View on Its Diagnostic, Prognostic, and Predictive Role. Int. J. Mol. Sci..

[44] Barth I., Schneider U., Grimm T., Karl A., Horst D., Gaisa N.T., Knüchel R., Garczyk S. (2018). Progression of urothelial carcinoma in situ of the urinary bladder: A switch from luminal to basal phenotype and related therapeutic implications. Virchows Arch..

[45] Gunia S., Koch S., Hakenberg O.W., May M., Kakies C., Erbersdobler A. (2011). Different HER2 protein expression profiles aid in the histologic differential diagnosis between urothelial carcinoma in situ (CIS) and non-CIS conditions (dysplasia and reactive atypia) of the urinary bladder mucosa. Am. J. Clin. Pathol..

[46] Dyrskjøt L., Zieger K., Kissow Lildal T., Reinert T., Gruselle O., Coche T., Borre M., Ørntoft T.F. (2012). Expression of MAGE-A3, NY-ESO-1, LAGE-1 and PRAME in urothelial carcinoma. Br. J. Cancer.

[47] Hodgson A., Jungbluth A.A., Katabi N., Xu B., Downes M.R. (2020). Evaluation of cancer testis antigen (CT10, PRAME) and MHC I expression in high-grade urothelial carcinoma of the bladder. Virchows Arch..

[48] Kaczorowski M., Chłopek M., Kruczak A., Ryś J., Lasota J., Miettinen M. (2022). PRAME Expression in Cancer. A Systematic Immunohistochemical Study of >5800 Epithelial and Nonepithelial Tumors. Am. J. Surg. Pathol..

[49] Kobayashi G., Hayashi T., Sentani K., Babasaki T., Sekino Y., Inoue S., Uraoka N., Hanamoto M., Nose H., Teishima J. (2022). Clinicopathological significance of claspin overexpression and its efficacy as a novel biomarker for the diagnosis of urothelial carcinoma. Virchows Arch..

[50] Arias-Stella J.A., Shah A.B., Gupta N.S., Williamson S.R. (2018). CK20 and p53 Immunohistochemical Staining Patterns in Urinary Bladder Specimens With Equivocal Atypia. Arch. Pathol. Lab. Med..

[51] Lawless M.E., Tretiakova M.S., True L.D., Vakar-Lopez F. (2018). Flat Urothelial Lesions With Atypia: Interobserver Concordance and Added Value of Immunohistochemical Profiling. Appl. Immunohistochem. Mol. Morphol..

[52] Hedegaard J., Lamy P., Nordentoft I., Algaba F., Høyer S., Ulhøi B.P., Vang S., Reinert T., Hermann G.G., Mogensen K. (2016). Comprehensive Transcriptional Analysis of Early-Stage Urothelial Carcinoma. Cancer Cell.

[53] Robertson A.G., Kim J., Al-Ahmadie H., Bellmunt J., Guo G., Cherniack A.D., Hinoue T., Laird P.W., Hoadley K.A., Akbani R. (2017). Comprehensive Molecular Characterization of Muscle-Invasive Bladder Cancer. Cell.

[54] Garczyk S., Bischoff F., Schneider U., Golz R., von Rundstedt F.C., Knüchel R., Degener S. (2021). Intratumoral heterogeneity of surrogate molecular subtypes in urothelial carcinoma in situ of the urinary bladder: Implications for prognostic stratification of high-risk non-muscle-invasive bladder cancer. Virchows Arch..

[55] Sanguedolce F., Zanelli M., Palicelli A., Ascani S., Zizzo M., Cocco G., Björnebo L., Lantz A., Falagario U.G., Cormio L. (2022). Are We Ready to Implement Molecular Subtyping of Bladder Cancer in Clinical Practice? Part 1: General Issues and Marker Expression. Int. J. Mol. Sci..

[56] Sanguedolce F., Zanelli M., Palicelli A., Ascani S., Zizzo M., Cocco G., Björnebo L., Lantz A., Landriscina M., Conteduca V. (2022). Are We Ready to Implement Molecular Subtyping of Bladder Cancer in Clinical Practice? Part 2: Subtypes and Divergent Differentiation. Int. J. Mol. Sci..

[57] Asgari M., Nabi Maybodi M., Abolhasani M. (2016). Differential diagnosis of urothelial carcinoma in situ from non-neoplastic urothelia: Analysis of CK20, CD44, P53 and Ki67. Med. J. Islam. Repub. Iran..

[58] Wullweber A., Strick R., Lange F., Sikic D., Taubert H., Wach S., Wullich B., Bertz S., Weyerer V., Stoehr R. (2021). Bladder Tumor Subtype Commitment Occurs in Carcinoma In Situ Driven by Key Signaling Pathways Including ECM Remodeling. Cancer Res..

[59] Yin H., He Q., Li T., Leong A.S. (2006). Cytokeratin 20 and Ki-67 to distinguish carcinoma in situ from flat non-neoplastic urothelium. Appl. Immunohistochem. Mol. Morphol..

[60] Babjuk M., Böhle A., Burger M., Capoun O., Cohen D., Compérat E.M., Hernández V., Kaasinen E., Palou J., Rouprêt M. (2017). EAU Guidelines on Non-Muscle-invasive Urothelial Carcinoma of the Bladder: Update 2016. Eur. Urol..

[61] Fernandez-Gomez J., Madero R., Solsona E., Unda M., Martinez-Piñeiro L., Gonzalez M., Portillo J., Ojea A., Pertusa C., Rodriguez-Molina J. (2009). Predicting nonmuscle invasive bladder cancer recurrence and progression in patients treated with bacillus Calmette-Guerin: The CUETO scoring model. J. Urol..

[62] Palou J., Rodríguez-Rubio F., Millán F., Algaba F., Rodríguez-Faba O., Huguet J., Villavicencio H. (2009). Recurrence at three months and high-grade recurrence as prognostic factor of progression in multivariate analysis of T1G2 bladder tumors. Urology.

[63] Solsona E., Iborra I., Dumont R., Rubio-Briones J., Casanova J., Almenar S. (2000). The 3-month clinical response to intravesical therapy as a predictive factor for progression in patients with high risk superficial bladder cancer. J. Urol..

[64] Cheng L., Cheville J.C., Neumann R.M., Leibovich B.C., Egan K.S., Spotts B.E., Bostwick D.G. (1999). Survival of patients with carcinoma in situ of the urinary bladder. Cancer.

[65] Hashizume A., Umemoto S., Yokose T., Nakamura Y., Yoshihara M., Shoji K., Wada S., Miyagi Y., Kishida T., Sasada T. (2018). Enhanced expression of PD-L1 in non-muscle-invasive bladder cancer after treatment with Bacillus Calmette-Guerin. Oncotarget.

[66] Woldu S.L., Gerald T., Margulis V., Halstuch D., Ber Y., Lifshitz K., Margel D., Lotan Y., Jia L. (2022). PD-L1 expression and BCG response in nonmuscle invasive bladder cancer. J. Clin. Oncol..

[67] Taylor A.S., Acosta A.M., Al-Ahmadie H.A., Mehra R. (2023). Precursors of urinary bladder cancer: Molecular alterations and biomarkers. Hum. Pathol..

[68] Sarkis A.S., Dalbagni G., Cordon-Cardo C., Melamed J., Zhang Z.F., Sheinfeld J., Fair W.R., Herr H.W., Reuter V.E. (1994). Association of P53 nuclear overexpression and tumor progression in carcinoma in situ of the bladder. J. Urol..

[69] Schmitz-Dräger B.J., van Roeyen C.R., Grimm M.O., Gerharz C.D., Decken K., Schulz W.A., Bültel H., Makri D., Ebert T., Ackermann R. (1994). P53 accumulation in precursor lesions and early stages of bladder cancer. World J. Urol..

[70] Shariat S.F., Kim J., Raptidis G., Ayala G.E., Lerner S.P. (2003). Association of p53 and p21 expression with clinical outcome in patients with carcinoma in situ of the urinary bladder. Urology.

[71] Akhtar M., Al-Bozom I.A., Ben Gashir M., Taha N.M., Rashid S., Al-Nabet A. (2019). Urothelial Carcinoma In Situ (CIS): New Insights. Adv. Anat. Pathol..

[72] Sato M., Yanai H., Morito T., Oda W., Shin-no Y., Yamadori I., Tshushima T., Yoshino T. (2011). Association between the expression pattern of p16, pRb and p53 and the response to intravesical bacillus Calmette-Guerin therapy in patients with urothelial carcinoma in situ of the urinary bladder. Pathol. Int..

[73] Hoffman-Censits J.H., Lombardo K.A., Parimi V., Kamanda S., Choi W., Hahn N.M., McConkey D.J., McGuire B.M., Bivalacqua T.J., Kates M. (2021). Expression of Nectin-4 in Bladder Urothelial Carcinoma, in Morphologic Variants, and Nonurothelial Histotypes. Appl. Immunohistochem. Mol. Morphol..

