# Supplementary figures and images for: Diagnostic, Prognostic, and Predictive Tissue Biomarkers in Urothelial Carcinoma In Situ: A Narrative Review
Source: Diagnostics (Basel). 2025 Aug 26;15(17):2163. doi: 10.3390/diagnostics15172163 (PMC12428764; doi:10.3390/diagnostics15172163)

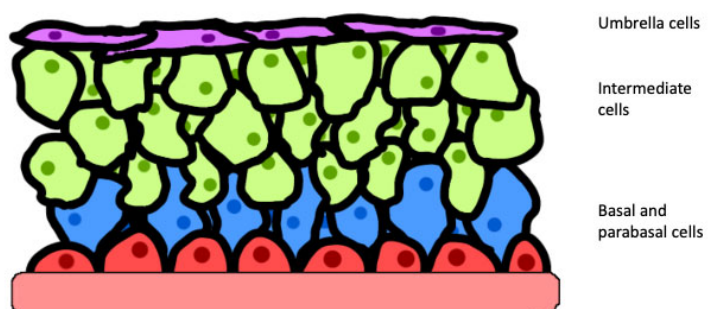

Figure S1: HE-stained histological section and diagram.

Supplement: Supplementary file 1 [file diagnostics-15-02163-s001.zip › diagnostics-3755848-supplementary.pdf]
